# Prolactin Expression in the Baboon (*Papio hamadryas*) Eye

**DOI:** 10.3390/ani12172288

**Published:** 2022-09-03

**Authors:** María Lourdes Garza-Rodríguez, Iram Pablo Rodríguez-Sanchez, Rafael González-Álvarez, Maricela Luna, Carlos Horacio Burciaga-Flores, Fernando Alcorta-Nuñez, Orlando Solis-Coronado, Víctor Manuel Bautista de Lucio, Genaro A. Ramírez-Correa, Oscar Vidal-Gutiérrez, Diana Cristina Pérez-Ibave

**Affiliations:** 1 Servicio de Oncología, Hospital Universitario “Dr. José Eleuterio González”, Universidad Autónoma de Nuevo León, Monterrey 64440, Mexico; 2 Laboratorio de Fisiología Molecular y Estructural, Facultad de Ciencias Biológicas, Universidad Autónoma de Nuevo León, San Nicolás de los Garza 66455, Mexico; 3 Facultad de Medicina, Universidad Autónoma de Guadalajara, Zapopan 45129, Mexico; 4 Departamento de Neurobiología Celular y Molecular, Instituto de Neurobiología, Universidad Nacional Autónoma de México, Juriquilla 76230, Mexico; 5 División de Anatomía Patológica, Hospital General “Dr. Manuel Gea González”, Secretaría de Salud, Ciudad de México 14080, Mexico; 6 Departamento de Microbiología y Proteómica Ocular, Instituto de Oftalmología “Fundación de Asistencia Privada Conde de Valenciana”, Ciudad de México 06800, Mexico; 7 Department of Molecular Science, University of Texas Health Rio Grande Valley, McAllen, TX 78550, USA

**Keywords:** prolactin (PRL), prolactin receptor (PRLR), *Papio hamadryas*, eye, retina, ganglion cell layer (GCL)

## Abstract

**Simple Summary:**

In primates, prolactin (PRL) is expressed in the pituitary gland; however, the extra-pituitary expression has been reported, including in the eye. The baboon is an excellent animal model for understanding the possible role of this hormone in the eye. The objective of this study was to detect PRL and its receptor expression in the eye tissues of fetal and adult baboons. Moreover, we performed *PRL* gene sequencing and a phylogenetic analysis to identify the evolutionary divergence in human and primate genes of *PRL*. We detected *PRL* and *PRLR* expression in all retinal cell lineages in fetal and adult baboons. No expression of these genes was detected in the cornea, lens, sclera, and iris. *PRL* and *PRLR* fit the hypothesis of evolutionary purifying gene selection. According to our findings, PRL has a role in prenatal and post-natal homeostasis of the baboon eye. We suggest that the hormone triggers autocrine and paracrine-specific functions in eye development.

**Abstract:**

Prolactin (PRL) is a hormone expressed in lactotrophs cells of the pituitary gland in primates. Extra pituitary expression of PRL has been reported, including the eye; however, expression in the developing eye of primates is limited. The aim of the study was determining the expression of *PRL* and *PRL* receptor (*PRLR*) (mRNAs and proteins) in adult and fetal baboon (*Papio hamadryas*) ocular tissues. Methods: We analyzed PRL and PRLR in baboon eyes tissues by immunofluorescence. The mRNAs of *PRL* and *PRLR* were detected by RT-PCR, cDNA was cloned, and sequenced. Furthermore, we performed a phylogenetic analysis to identify the evolutionary forces that underlie the divergence of *PRL* and *PRLR* primate genes. Results: We observed the expression of PRL and PRLR (mRNAs and proteins) in all retinal cell lineages of fetal and adult baboon. *PRL* and *PRLR* fit the hypothesis of evolutionary purifying gene selection. Conclusions: mRNA and protein of PRL and PRLR are expressed in fetal and adult baboon retinal tissue. PRL may trigger autocrine and paracrine-specific actions in retinal cell lines.

## 1. Introduction

Human prolactin hormone (PRL) is a 23 kDa protein of 199 aa residues [1]. *PRL* gene has 5 exons and 4 introns, and is located on chromosome 6 [2]. PRL is an evolutionarily ancient hormone, that serves as a molecular correlate of seasonal timing in most species [3]. PRL belongs to a gene family that comprises PRL, growth hormone, and somatolactin [4]. In mammals, PRL evolved very slowly, but this near-stasis was interrupted by bursts of rapid change during the evolution of primates, this change is associated with the biological function of the hormone [5,6].

PRL is a pleiotropic neurohormone expressed by lactotrophs cells of the anterior pituitary gland, from which prolactin is released into the bloodstream to act as a multifunctional hormone [4,7,8]. PRL promotes mammary gland development and lactation [2,9,10]. Other functions include: osmoregulation, growth, energy metabolism, immune response, brain function, and reproduction [11,12,13]. Moreover, PRL can both stimulate and inhibit blood vessel growth, dilation, permeability, and cell survival [14,15]. It is expressed in multiple extra-pituitary tissues in mammals such as the eye, ovary, breast, immune cells, testicle, brain, skin, etc. It may have autocrine or paracrine activity [8,16,17,18,19].

In rat, PRL is produced in retina, and in canines a retinal protective role was reported [20,21]. In humans, PRL has antioxidant activity in the retina by reducing oxidative stress and regulating glutathione levels [22]. In diabetic patients, PRL acquires antiangiogenic properties upon its proteolytic cleavage to vasoinhibin, a PRL fragment that inhibits angiogenesis, vascular permeability, and vasodilation [18,23,24].

Overexpression of PRL in patients with diabetic retinopathy results in accumulation of vasoinhibins in the retina, inhibition of vascular endothelial growth factor (VEGF), inactivation of nitric oxide synthase (NOS), and prevention of retinal vasopermeability [23,25,26,27,28,29]. Therefore, it is essential to understand the role of PRL and its isoforms in retinal tissues.

Due to the morphological, molecular, and phylogenetic relationship with humans, primates and rodents have been recommended as ideal animal models to study ocular tissues [30]. The baboon (*Papio hamadryas*) is an excellent animal model to understand eye development, physiology, and pathological ocular conditions, as diabetic retinopathy [21].

## 2. Materials and Methods

### 2.1. Animal Model

We analyze baboons frozen eyes obtained from the Texas Biomedical Research Institute (TBRI) Biobank of San Antonio, TX, USA. Animal procedures were performed according to ethical guidelines and were reviewed by the Institutional Animal Care and Use Committee of the TBRI of San Antonio, TX, USA. All the animals shared the same diet and environmental conditions before sample collection. All baboons were gang-housed and fed *ad libitum* on a standard low-fat chow diet (Harlan Tecklad 15% Monkey Diet, 8715, Indianapolis, IN, USA). Pregnant baboons underwent a caesarean section between days 136–139 of gestation. Animal feeding schedules, environmental conditions, and sample collection were described previously [31].

### 2.2. Biological Material

Nitrogen frozen ocular and pituitary tissues from TBRI biobank belong to seven adult female baboons (*Papio hamadryas*) ranging between 7 and 40 years of age and two four-month-old fetuses. One ocular piece was frozen in liquid nitrogen, while the other was included in 4% paraformaldehyde for immunofluorescence (IF) assays. A detailed description of the analyzed tissues is provided in Supplementary Materials (Table S1).

### 2.3. RNA Isolation of Ocular Tissues

Each ocular piece was dissected, and the cornea, iris, lens, sclera, retina-RPE-choroid, and optic nerve were separated from both adult and fetus baboon eyes. The eyes were thawed in the cold at 4 °C for 15–20 min before dissection. Both dissection and thawing of ocular tissues were performed on ice. Total RNA was extracted with TRIzol™ reagent (Invitrogen, Carlsbad, CA, USA), according to manufacturer instructions. The protocols to analyze RNA integrity and purity for molecular analyses have been described previously [31].

### 2.4. Reverse Transcription (RT) and Polymerase Chain Reaction (PCR)

Retrotranscription (RT) reactions were described above [21]. For PCR reactions, we designed consensus PRL-primers using previously *PRL*-reported sequences from *Macaca mulatta* and *Homo sapiens* [Gene Bank access number: NM_001047128.1 and NM_000948, respectively]. The forward primer (5′-GTGAAGTGTGTTTCCCTGCAA-3′) was designed to initiate amplification of 11 bases upstream from the translation initiation codon and the reverse primer (5′-TGCTTGGGTGTAACAGGTCTC-3′) to hybridize 89 bases after the termination codon.

The amplification reaction was carried out using 500 ng of cDNA (from RT reaction), 10 µM of each primer, PCR Master Mix 2x from Promega (Fitchburg, WI, USA), and a final volume of 25 µL in a Veriti^®^ 96-Well Thermal Cycler (Thermo-Fisher Scientific, Waltham, MA, USA). The amplification programs initial hold was at 94 °C for 3 min, followed by 32 cycles, each including incubations at 94 °C for 30 s, 60 °C for 45 s and 72 °C for 1 min and 30 s, and a final elongation step at 72 °C for 6 min. The amplification products were visualized on 1% agarose gels stained with ethidium bromide under UV light.

### 2.5. Molecular Cloning, Sequencing, and Sequence Analysis of PRL mRNA

Positive clones were sequenced using the Big Dye terminator cycle sequencing kit v3.1 with specific primers and/or M13 universal primers. The amplified products were cloned in 3.5-kb XL-TOPO vector and transformed into electrocompetent *E. coli* strain Top 10 according to the manufacturer’s specifications (Invitrogen). The reactions were analyzed in the ABI PRISM 3100 Genetic Analyzer using Sequencing Analysis Software v5.3 (Applied Biosystems, Foster City, CA, USA). The sequences obtained from clones were aligned with the human PRL orthologous gene [GenBank: NM_000948.5] using the CLUSTAL W2 program [32], followed by manual corroboration. Novel sequences were deposited in the GenBank database (Accession number HM103903) [32].

### 2.6. Phylogenetic Analysis

Phylogenetic tree was constructed with the sequences of *PRL* cDNA and the reported sequence of *PRLR* in NCBI web page (XM_003899569). For *PRLR,* we used the Olive baboon (*Papio anubis*) sequence because the *Papio hamadrayas* sequence was not available in the NCBI web page. We predicted amino acidic sequences, and the phylogenetic trees were built with MEGA 6.06 software (Molecular Evolutionary Genetics Analysis. Version 6.0.6. Pennsylvania State University PA, USA ) [33]. We use the Neighbor-Joining (NJ) method; then, a bootstrap test was carried out with 1000 replicates [27]. Sequences used in this study are listed in Supplementary Materials Table S2. We tested the hypothesis of positive or adaptive evolution (dN > dS), purifying selection (dN < dS), and neutrality (dN = dS) to identify the evolutionary forces that underlie the process of divergence in the PRL and PRLR primate genes. We calculated the non-synonymous (causes an amino acid change) dN and synonymous (does not cause an amino acid change) dS distances, respectively, by the Li-Wu-Luo method (Kimura 2-parameters) [28] from PRL and PRLR coding sequences from apes, OWM (old world monkeys) and NWM (new world monkeys) with their lemur counterpart. Second, we tested whether dN is significantly ≤, =, or ≤dS using a codon-based Z selection test as implemented in MEGA 6.06 software [33]. Differences were considered statistically significant at a *p* < 0.05.

### 2.7. IF Assays

Ocular tissues were fixed in 4% paraformaldehyde and treated as we described previously [29]. The primary antibodies were incubated overnight at 4 °C, a mouse polyclonal anti-human PRL antibody (PA1-85812, Pierce) at a dilution 1:100, and a mouse monoclonal anti-human PRLR antibody (MA1-610, Pierce) at a dilution 1:250. All the antibodies were diluted in T-TBS buffer 1% skim milk. The specificity of staining was determined by adsorbing the PRL primary antibody with excess recombinant human PRL (Norditropin, PiSA, Guadalajara, Mexico) at a 1 mg/ml concentration. The primary antibody was omitted as a negative control. Some eye sections (5 μm-thick) were double-stained with anti-PRL and anti-PRLR antibodies. After mouse anti-PRL incubation, the slides were incubated with a mouse on mouse blocking reagent (MKB-2213, Vector Laboratories, Newark, CA, USA) for 1 h. The slides were washed two times for 5 min with TBS; afterward, the slides were incubated with an anti-PRLR antibody overnight at room temperature. After primary antibodies incubation, slides were washed five times with T-TBS buffer for 10 min. Then sections were incubated for 2 h in darkness at room temperature with secondary antibodies Cy3™ goat anti-rabbit IgG (A10520, Life Technologies Carlsbad, CA, USA)(dilution 1:4000), Cy5™ goat anti-rabbit IgG (A10523, Life Technologies) (dilution 1:4000) and goat anti-mouse IgG FITC (62-6511, Life Technologies) (dilution 1:250). The sections were washed five times with T-TBS, and counterstained with DAPI (D-9542, Sigma Aldrich, Saint Louis, MO, USA) at 0.1 µg/ml. The sections were washed and mounted with Vectashield (H-1000, Vector Laboratories) and examined using an LSM 780 confocal microscope (Carl Zeiss, Germany) using lasers at excitation wavelengths of 550 nm (Cy3™), 650nm (Cy5™), and 495 nm (FITC). Digital images were equally balanced in brightness and contrast.

## 3. Results

### 3.1. PRL mRNA Is Expressed in the Baboon Eye Retina

**Figure 1:**
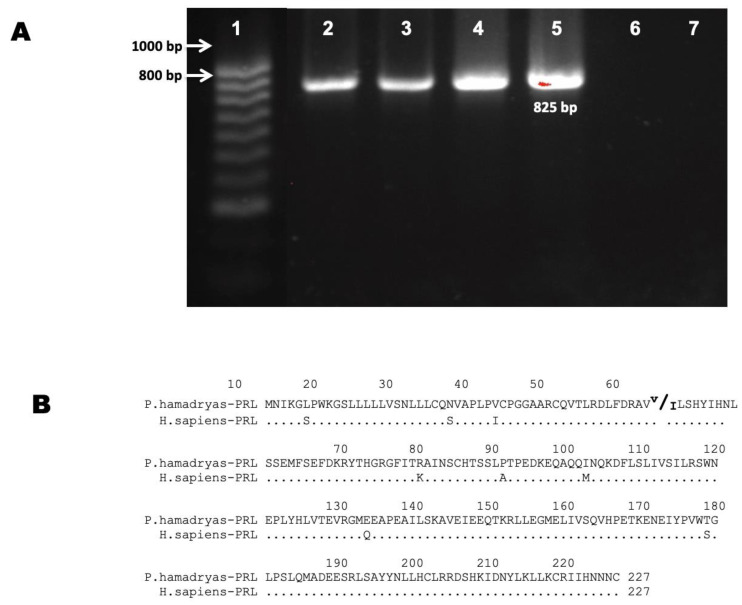
Prolactin transcript’s expression in baboon eye. (**A**) Agarose gel electrophoresis of PCR products obtained with primers for cDNA of *PRL* generated from different retinal tissues. Lanes: (1) Molecular weight marker, (2) baboon adult retina, (3) baboon fetal retina, (4) baboon fetal pituitary, (5) baboon adult pituitary as a positive control, (6) baboon adult liver as a negative control, and (7) Non-Template Control (NTC). (**B**) Sequence of the PRL predicted from the open reading frame according to sequenced amplicons and comparison with the human *PRL* sequence.

We analyzed seven different baboon tissues by RT-PCR: five of ocular origin, one from pituitary (positive control), and one from liver (negative control) (Figure 1). Baboon tissues used in this study are described in Supplementary Materials Table S1. The PCR controls (positive and negative) showed the expected results. The eye tissues expressed mRNAs for *PRL* (Table 1). *PRL* cDNA was detected in fetal and adult retinal tissues. The *PRL* amplicons were of 825 pb as expected (Figure 1A). In one sample, we found two different types of *PRL* mRNAs (expression of two different gene alleles) (Figure 1), the same transcripts were found in the pituitary tissue of the same animal.

We detected *PRL* expression (mRNAs) in all the retina tissues analyzed (Table 1). However, no expression was detected in other ocular tissues (cornea, lens, sclera, and iris).

The PCR products from *PRL* cDNAs were cloned and sequenced. Their analyses showed the characteristic organization of mRNAs of this gene class: exon I: 547 bp, exon II = 175 bp, exon III = 107 bp, exon IV = 179 bp, and exon V = 347 bp [11]. To determine the polypeptides encoded by the baboon *PRL* cDNAs, open reading frames were identified, and the sequence was translated in silico. The complete amino acid sequence is shown in Figure 1B. The predicted amino acidic sequence corresponds to a hormone of 227 amino acids (aa). One of the specimens showed a change from valine to isoleucine in amino acid 52 (Figure 1B). *PRL* transcripts isolated from the baboon eye are identical to their baboon pituitary counterpart, which confirms a new expression site for this hormone in the baboon.

### 3.2. PRL and PRLR Protein Expression in Fetus and Adult Baboon’s Retina

**Figure 2:**
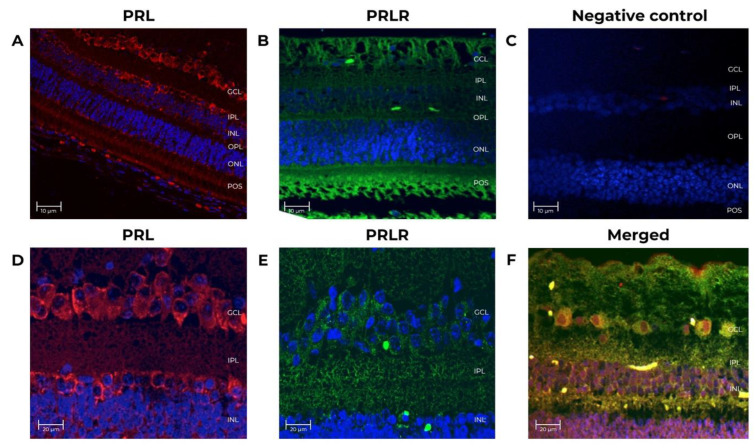
Prolactin and prolactin receptor immunoreactivity in the neural retina from adult female baboons. The tissues correspond to adult baboon ID 14871. Confocal images of immuno-stained retina sections identified cells expressing PRL and PRLR. (**A**) PRL expression (red, 1st Ab: mouse anti-human PRL 1:100; goat anti-mouse IgG Cy5 1:400). Nuclei (blue) were counterstained with DAPI. (**B**) PRLR expression (green, 1st Ab: mouse anti-human PRLR 1:250; goat anti-mouse IgG-FITC 1:250), nuclei (blue). (**C**) Negative control for IgG Cy3 antibody has no primary antibody (mouse anti-human PRL 1:100). (**D**) PRL retina sections (5 μm-thick) at 25X magnification, scale-bar 20 μm. (**E**) PRLR sections at 25X magnification, scale-bar 20 μm. (**F**) Double-stained sections identified cells expressing PRL (red, 1st Ab: mouse anti-human PRL 1:100; goat anti-mouse IgG Cy5 1:4000), and PRLR (green, 1st Ab: mouse anti-human PRLR 1:250; goat anti-mouse IgG-FITC 1:250) at 25X, scale-bar 20 μm. GCL (ganglion cell layer), INL (inner nuclear layer), ONL (outer nuclear layer), IPL (inner plexiform layer), OPL (outer plexiform layer), and POS (photoreceptor outer segment).

**Figure 3:**
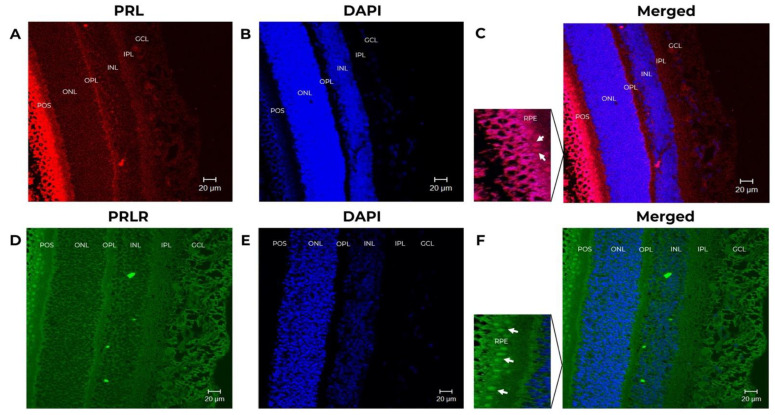
Prolactin (PRL) and prolactin receptor (PRLR) immunoreactivity in the neural retina in baboon fetus. The tissues correspond to fetus baboon ID 12354. Confocal images of immuno-stained retina sections identified cells expressing PRL and PRLR. (**A**) PRL expression (red, 1st Ab: mouse anti-human PRL 1:100; goat anti-mouse IgG Cy5 1:4000) in the retina of baboon fetus. (**B**) Stained retina sections with DAPI to counterstain nuclei (blue) at 25X magnification, scale-bar 20 μm. (**C**) Merged images of stained retinal sections to show PRL expression and DAPI, at 25X magnification, scale-bar 20 μm. (**D**) Cells expressing PRLR (green, 1st Ab: mouse anti-human PRLR 1:250; goat anti-mouse IgG-FITC 1:250). In the magnification the expression of PRL is positive (white arrows indicate the PRL positive cells). (**E**) Retinal sections stained with DAPI (nuclei in blue) at 25X magnification, scale-bar 20 μm. (**F**) Merged images to show PRLR expression and DAPI, at 25X magnification, scale-bar 20 μm. In the magnification the expression of PRLR is positive (white arrows indicate the PRLR positive cells) GCL (ganglion cell layer), INL (inner nuclear layer), ONL (outer nuclear layer), IPL (inner plexiform layer), OPL (outer plexiform layer), RPE (retinal pigmented epitelium), and POS (photoreceptor outer segment).

IF analysis showed the localization and distribution of PRL and PRLR proteins in the retina of 4-month-old fetuses and 15-year-old non-pregnant female adult baboons (Figure 2 and Figure 3).

**Figure 4:**
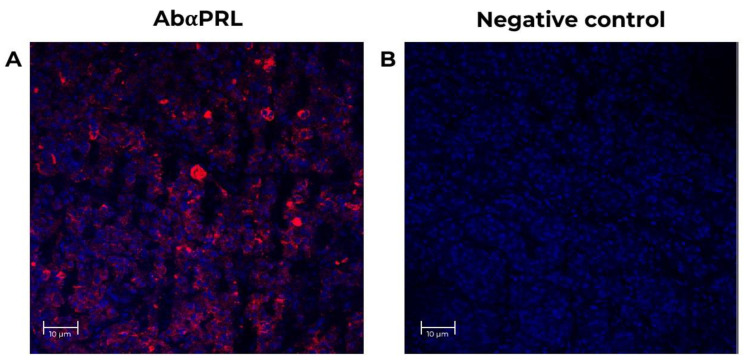
Positive control for specificity PRL antibodies in the neural retina from adult female baboons. The tissues correspond to fetus baboon ID 12354. (**A**) Confocal images of stained baboon adult pituitary sections to identify cells expressing PRL (red, 1st Ab: mouse anti-human PRL 1:100; goat anti-mouse IgG Cy5 1:4000) in lactotrophs adult baboon pituitary. Nuclei (blue) were labeled with DAPI. (**B**) Negative control with no primary antibody (mouse anti-human PRL 1:100, red signal) in baboon pituitary slices, at 25X magnification, scale-bar 10 μm.

A baboon pituitary control was run to validate the techniques and antibodies. As shown in Figure 4, an intense PRL (red) immunoreactivity was detected within the cytoplasm of adult baboon lactotrophs [34]. No IF signal was detectable when the primary antibodies were omitted (Figure 4B). In adult baboon retina, PRL and PRLR were detected in the rod and cones layer, the inner plexiform layer/outer plexiform layer (IPL/OPL), the outer nuclear layer (ONL), inner nuclear layer (INL), the photoreceptor outer segments (POS), and in the ganglion cell layer (GCL). The GCL cells showed the highest IF signal.

PRL and PRLR were detected in the IPL, INL, OPL, ONL, POS, and the pigmented epithelium (RPE) in the fetus baboon’s retina. The signal from POS and RPE (Figure 3A) showed the highest IF signal. In baboon fetus, there is an appearance of a multilayer of the RPE because there is no complete differentiation of the pigment epithelium since the baboon fetus eye has been reported to continue to undergo cell differentiation up to 23 weeks of gestation [30]. The analyzed images are of fetuses of approximately 4 months of gestation (17 weeks of gestation). The images were reviewed by a pathologist expert in retina.

In summary, we detected the expression of PRL and PRLR in all retinal cell layers. The most abundant signal was in RPE, POS, and GCL. In support of the specificity of these findings, no staining was observed when the primary antibodies were omitted or when the anti-PRL antibody was preadsorbed with recombinant human PRL.

### 3.3. Phylogenetic Analysis Demonstrates Gene Purifying Selection

**Figure 5:**
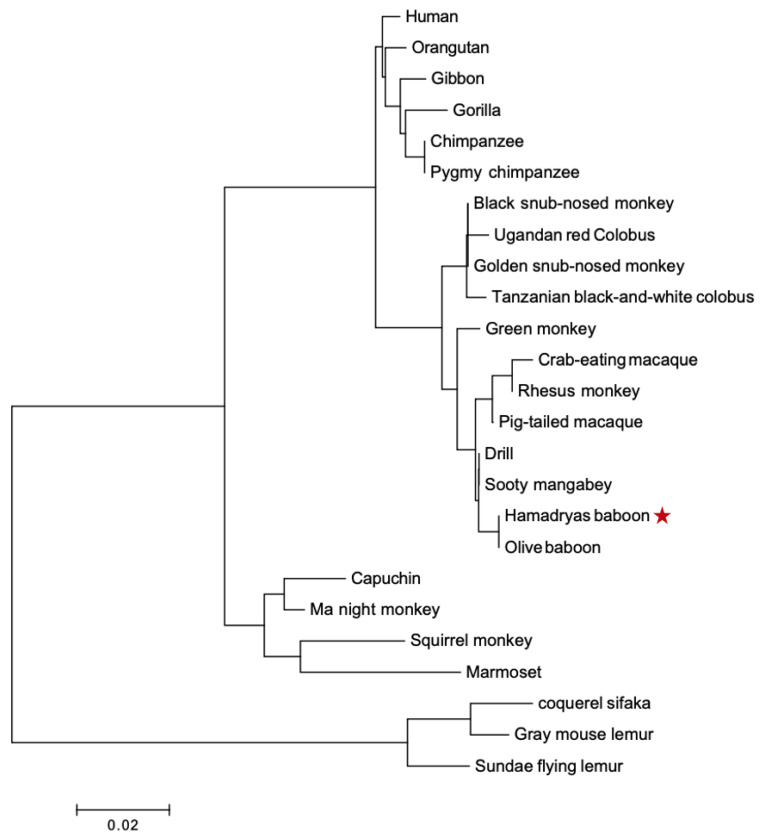
Phylogenetic tree of PRL proteins from various primates. Clades are specific in linage, apes, OWM, NWM, lemur, and out-group. The tree was built using MEGA version 6.06 by the NJ method and further bootstrap analysis of 1000 replicas. The red star corrrespon to the sequence we found.

**Figure 6:**
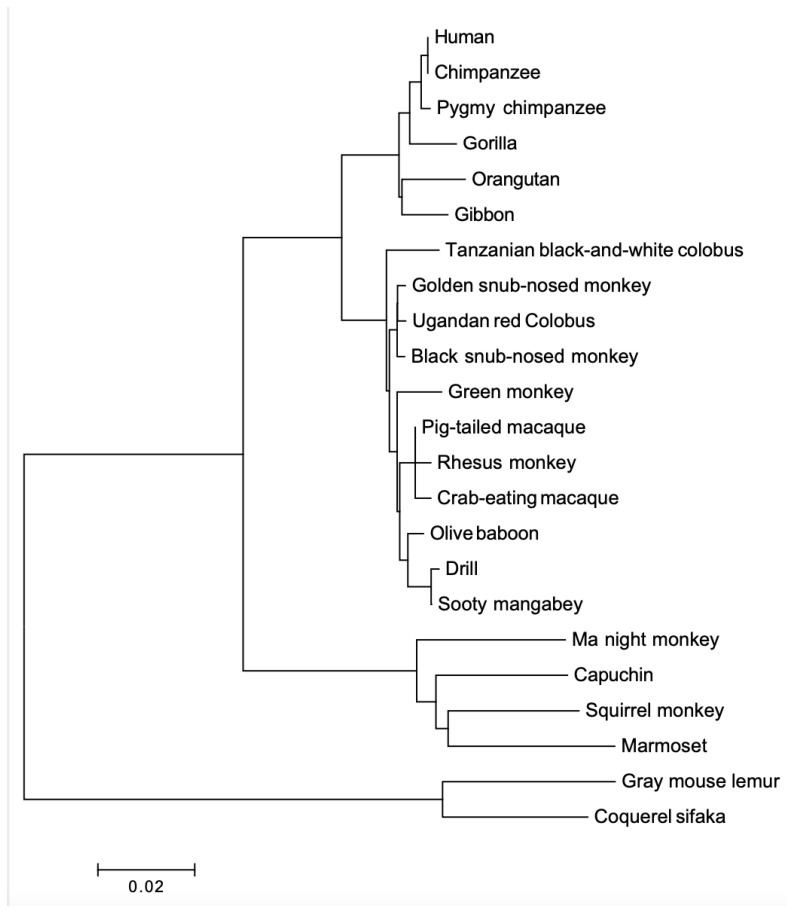
Phylogenetic tree of PRLR proteins from various primates. The tree was built using MEGA version 6.06 by the NJ method and further bootstrap analysis of 1000 replicas. Clades are in linage specific manner.

Two phylogenetic trees were built for *PRL* and *PRLR*. The *PRL* tree (Figure 5) shows four clades in a lineage-specific manner, and they correspond to apes, OWM, NWM, and lemur (out-group). It confirms the orthology between primate *PRL* genes. The *PRLR* tree (Figure 6) shows the same clades. We confirmed that *PRL* and *PRLR* evolution fit the hypothesis of purifying selection (d_N_ < d_S_, *p* < 0.05).

## 4. Discussion

The present study demonstrates the expression of PRL and PRLR’s mRNA, and their corresponding proteins in the retina of fetuses and adult baboons. The PRL transcripts isolated from the baboon eye, were identical to their baboon pituitary counterparts, which confirms that the retina expresses this hormone and its receptor [11,12,13,14,16,17,18]. The *PRL* cDNAs was detected only in the retina, and there was no expression in the cornea, lens, sclera, and iris. This finding agrees with the reports in green monkeys and rats, where PRL and PRLR’s mRNA and protein are localized in the retina [17,19].

The retina is a highly organized laminar structure of specialized neural cells at the back of the eye. Diseases such as retinitis pigmentosa, age-related macular degeneration, diabetic retinopathy, and glaucoma can lead to blindness due to the loss of function of these specialized cell types [35]. The retina produces biological factors that act in an autocrine or paracrine manner to regulate the function of the neural cells and blood vessels to provide normal vision [19]. There has been proposed that PRL is one of those essential biological factors for retinal homeostasis, and our finding supports this hypothesis [22,36]. Additional studies are needed to confirm the function of PRL in retinal microenvironment and its implications in the ocular health and diseases.

The prediction of the *PRL* amino acid sequences from the retina’s cDNA (Figure 1) revealed the presence of two molecular species in one sample, probably because of the expression of two different alleles also found in the pituitary. *PRL* polymorphisms have been reported in association with several phenotypic traits, including: milk production in bovines [37], egg production in chickens [38], reproduction [39], abortion [40], and weight [41].

PRL and PRLR immunostaining co-localization shows the distribution in the RPE, ONL, OPL, INL, IPL, and GCL (Figure 2C,D). Using IF, we demonstrated that all retinal cell layers express PRL and PRLR. The most abundant expression was in RPE and POS. This PRL and PRLR expression pattern agrees with previous reports in rats and green monkeys, where the PRL signal is detected throughout the retina layers. However, to our knowledge, this is the first report on baboons. The similarity of PRL with PRLR distribution indicates that these proteins are produced by the same or neighbor retinal cells. The distribution of PRL and PRLR is probably conserved in mammals. Rivera et al. also reported the presence of mRNA of *PRL* and its receptor in these retinal cell lines by in situ hybridization [19].

Furthermore, the presence of this hormone and its receptor in fetuses and adult baboons may indicate that PRL participates in baboon eye development. PRL-derived peptides (vasoinhibins) act on endothelial cells to inhibit blood vessel growth and dilation and promote apoptosis-mediated vascular regression [15,42]. Although, there are no studies showing if the retinal cell lines that express PRL also express vasoinhibins, the presence of the hormone can be interpreted as evidence that these peptides could be generated by proteolysis of PRL [18,28]. This theory is supported by reports demonstrating that the retinal expression of PEDF (pigmented epithelium-derived factor) in RPE and GCL acts as an antiangiogenic factor [30,31,34,43,44]. A novel function of vasoinhibins as inhibitors of the increased retinal VEGF-induced vasopermeability associated with diabetic retinopathy suggests that vasoinhibins can be developed as new therapeutic agents to control the excessive retinal vasopermeability observed in diabetic retinopathy and other vasoproliferative retinopathies [23,25,26,31]. Inhibition of PRL synthesis in zebrafish reduced eye size, implicating this hormone’s role in eye development [35,45]. Therefore, PRL could be explored as a therapeutic agent for patients with diabetic retinopathy or any other disease that involves an alteration in ocular angiogenesis. However, further studies are required.

We estimated the dN and dS rates in the PRL and PRLR coding regions and found that they fit the hypothesis of purifying of selection (dN < dS) for both cDNAs. Purifying of selection is an indicator of functional genes. As previously described, the changes during the evolution of primates, are associated with the biological function of the hormone [5,6].

## 5. Conclusions

In conclusion, we show that most cell types of baboon retina produce PRL and PRLR. Our findings suggest that PRL may trigger autocrine and paracrine-specific actions in different retinal cell lines and that this hormone has prenatal and post-natal essential functions in the baboon. *PRL* and *PRLR* evolution is an indicator that both genes are essential in development and homeostasis in primates.

## Figures and Tables

**Table 1:** Tissue distribution of *PRL* mRNAs and proteins in baboon eye.

Condition	mRNA	Tissue						
		Retina	Cornea	Lens	Sclera	Iris	Pituitary (Positive Control)	Liver (Negative Control)
Adult	*PRL*	+	-	-	-	-	+	-
Condition	Protein	Cell type or layer (of retina)						
		Pigmented epithelia	Rods and cones	Outer nuclear layer	Outer plexiform layer	Inner nuclear layer	Inner plexiform layer	Ganglion cell layer
Adult	PRL	+++	+++	++	+	++	++	++
	PRLR	+++	+++	+	++	+	++	++
Fetus	PRL	+++	+++	++	++	++	++	++
	PRLR	+++	+++	+	++	+	++	++

The intensity of the fluorescent signal was qualitatively assessed on a + basis. A low signal was indicated by one +, intermediate signal by ++, and high signal intensity by +++.

## Data Availability

The novel sequence of *Papio hamadryas* prolactin (PRL) mRNA (complete cds) was deposited in the public GenBank database at https://www.ncbi.nlm.nih.gov/nuccore/HM103903, accessed on 6 December 2021. The phylogenetic analysis (MEGA program https://www.megasoftware.net/, accessed on 10 January 2022) in this study was a re-analysis of existing data, sequences used are openly available at the GenBank database at https://www.ncbi.nlm.nih.gov/, accessed on 10 February 2022. The mRNA and protein sequence accession numbers are listed in Supplementary Materials Table S2.

## Supplementary Materials

The following supporting information can be downloaded at: https://www.mdpi.com/article/10.3390/ani12172288/s1. Table S1: Characteristics of baboon tissue samples; Table S2: Sequences used in the phylogenetic analysis.
