# Supplementary material for: Prolactin Expression in the Baboon (Papio hamadryas) Eye
Source: Animals (Basel). 2022 Sep 3;12(17):2288. doi: 10.3390/ani12172288 (PMC9455022; doi:10.3390/ani12172288)
Supplement: Supplementary file 1 [file animals-12-02288-s001.zip › animals-1779930-supplementary.pdf]

animals

Supplementary Materials

Prolactin Expression in the Baboon (*Papio hamadryas*) Eye

María Lourdes Garza-Rodríguez, Iram Pablo Rodríguez-Sanchez, Rafael González-Álvarez, Maricela Luna, Carlos Horacio Burciaga-Flores, Fernando Alcorta-Nuñez, Orlando Solis-Coronado, Víctor Manuel Bautista de Lucio, Genaro A. Ramírez-Correa, Oscar Vidal-Gutiérrez and Diana Cristina Pérez-Ibave

Table S1. Characteristics of baboon tissue samples.

Animal ID	Tissue	Gender (M/F)	Age (years)
15607	Eye	Female	17
14871	Eye	Female	15
27845	Eye	Female	7
27927	Eye	Female	7
28347	Eye	Female	7
1X2942	Pituitary	Female	34
1X1962	Pituitary	Female	40
2X0281	Pituitary	Female	30
1X2199	Pituitary	Female	35
1X1927	Pituitary	Female	36
2X0209	Pituitary	Female	32
1X2574	Pituitary	Female	35
13965	Pituitary, eye, liver, and placenta	Pregnant female	18
12354	Pituitary, eye, and liver	Fetus	4 months
16248	Pituitary, eye, and liver	Fetus	4 months

Table S2. Sequences used in the phylogenetic analysis

	Accession No.			
	PRL		PRLR	
**Species**	**mRNA**	**Protein**	**mRNA**	**Protein**
		**Apes**		
Human (*Homo sapiens*)	V00566	CAA23829	M31661	AAA60174
Chimpanzee (*Pan troglodytes*)	XM_009450584	XP_009448859	XM_001150064	XP_001150064
Pygmy chimpanzee (*Pan paniscus*)	XM_008976790	XP_008975038	XM_003806664	XP_003806712
Orangutan (*Pongo abelii*)	XM_024248232	XP_024104000	NM_001159791	NP_001153263
Gorilla (*Gorilla gorilla gorilla*)	XM_004043332	XP_004043380	XM_004058975	XP_004059023
Gibbon (*Nomascus leucogenys*)	XM_012509062	XP_012364516	XM_003274943	XP_003274991
		**OWM**		
Olive baboon (*Papio anubis*)	XM_003897114	XP_003897163	XM_003899569	XP_003899618
Hamadryas baboon (*Papio hamadryas*)	HM103903	ADG56475	Not available	Not available
Sooty mangabey (*Cercocebus atys*)	XM_012031434	XP_011886824	XM_012055160	XP_011910550
Drill (*Mandrillus leucophaeus*)	XM_011998167	XP_011853557	XM_011969112	XP_011824502
Rhesus monkey (*Macaca mulatta*)	NM_001047128	NP_001040593	XM_001092241	XP_001092241
Pig-tailed macaque (*Macaca nemestrina*)	XM_011742657	XP_011740959	XM_011726668	XP_011724970
Crab-eating macaque (*Macaca fascicularis)*	XM_005553930	XP_005553987	XM_005556706	XP_005556763
Green monkey (*Chlorocebus sabaeus*)	XM_007973568	XP_007971759	XM_007961343	XP_007959534
Ugandan red Colobus (*Piliocolobus tephrosceles*)	XM_023209759	XP_023065527	XM_023216524	XP_023072292
Golden snub-nosed monkey (*Rhinopithecus* [illegible])	XM_010354096	XP_010352398	XM_010384082	XP_010382384
Black snub-nosed monkey (*Rhinopithecus bieti*)	XM_017873099	XP_017728588	XM_017894520	XP_017750009
Tanzanian black-and-white colobus (*Colobus angolensis palliatus*)	XM_011962769	XP_011818159	XM_011944340	XP_011799730
		**NWM**		

Capuchin (*Cebus capucinus imitator*)	XM_017544082	XP_017399571	XM_017548276	XP_017403765
Ma’s night monkey (*Aotus nancymaae*)	XM_012458516	XP_012313939	XM_012459967	XP_012315390
Squirrel monkey (*Saimiri boliviensis boliviensis*)	XM_003927308	XP_003927357	XM_003925930	XP_003925979
Marmoset (*Callithrix jacchus*)	XM_002746181	XP_002746227	XM_008992156	XP_008990404
		**Lemurs**		
Gray mouse lemur (*Microcebus murinus*)	XM_012753690	XP_012609144	XM_020290061	XP_020145650
Coquerel’s sifaka (*Propithecus coquereli*)	XM_012638648	XP_012494102	XM_012640511	XP_012495965
		**Out-group**		
Sundae flying lemur (*Galeopterus variegatus*)	XM_008593845	XP_008592067	Not available	Not available
